# Supplementary material for: The effect of caloric restriction on the increase in senescence-associated T cells and metabolic disorders in aged mice
Source: PLoS One. 2021 Jun 18;16(6):e0252547. doi: 10.1371/journal.pone.0252547 (PMC8213184; doi:10.1371/journal.pone.0252547)
Supplement: S1 Fig — Representative flow cytometric analysis of PD-1+ CD44+ T cells (PD-1+ MP T cells) from the spleen (A) and eVAT (B). (C) Representative flow cytometric analysis of CD11b+ F4/80+ macrophages; M1 type (CD11b+ F4/80+ CD11c+ CD206-) and M2 type (CD11b+ F4/80+ CD11c- CD206+) macrophages from eVAT. (DOCX) [file pone.0252547.s001.docx]

**S1 Fig.** **Gating strategy of immune cell populations in the stromal vascular fraction (SVF).**

Representative flow cytometric analysis of PD-1^+^ CD44^+^ T cells (PD-1^+^ MP T cells) from the spleen (**A**) and eVAT (**B**). (**C**) Representative flow cytometric analysis of CD11b^+^ F4/80^+^ macrophages; M1 type (CD11b^+^ F4/80^+^ CD11c^+^ CD206^-^) and M2 type (CD11b^+^ F4/80^+^ CD11c^-^ CD206^+^) macrophages from eVAT.
