# Supplementary material for: The effect of caloric restriction on the increase in senescence-associated T cells and metabolic disorders in aged mice
Source: PLoS One. 2021 Jun 18;16(6):e0252547. doi: 10.1371/journal.pone.0252547 (PMC8213184; doi:10.1371/journal.pone.0252547)
Supplement: S1 File — (DOCX) [file pone.0252547.s002.docx]

Figure 1

1. Body weight (g)

|  | Mean | SE |
| --- | --- | --- |
| Young | 22.27 | 0.24 |
| Aged | 38.90 | 0.57 |

eVAT (mg)

|  | Mean | SE |
| --- | --- | --- |
| Young | 350.2 | 41.7 |
| Aged | 1039.6 | 56.9 |

B. OGTT (mg/dl)

|  | 0 min | | 15 min | | 30 min | | 60 min | | 120 min | |
| --- | --- | --- | --- | --- | --- | --- | --- | --- | --- | --- |
|  | Mean | SE | Mean | SE | Mean | SE | Mean | SE | Mean | SE |
| Young | 85.5 | 3.2 | 342.5 | 9.1 | 290.7 | 23.9 | 180.7 | 12.1 | 101.7 | 4.5 |
| Aged | 90.0 | 2.2 | 379.3 | 15.9 | 334.7 | 32.3 | 266.7 | 23.5 | 163.3 | 13.7 |

ITT (% change)

|  | 0 min | | 15 min | | 30 min | | 60 min | | 120 min | |
| --- | --- | --- | --- | --- | --- | --- | --- | --- | --- | --- |
|  | Mean | SE | Mean | SE | Mean | SE | Mean | SE | Mean | SE |
| Young | 100 | 0 | 40.6 | 9.4 | 29.7 | 14.4 | 29.7 | 15.3 | 33.6 | 5.4 |
| Aged | 100 | 0 | 88.3 | 7.3 | 66.5 | 11.8 | 72.1 | 19.8 | 48.9 | 11.2 |

C. Insulin (ng/ml)

|  | Mean | SE |
| --- | --- | --- |
| Young | 0.38 | 0.015 |
| Aged | 0.49 | 0.025 |

Adiponectin (μg/ml)

|  | Mean | SE |
| --- | --- | --- |
| Young | 23.86 | 2.74 |
| Aged | 11.93 | 2.38 |

Leptin (ng/ml)

|  | Mean | SE |
| --- | --- | --- |
| Young | 0.98 | 0.11 |
| Aged | 6.53 | 2.34 |

Figure 2

B. eVAT p21 (%)

|  | CD3 | | CD4 | | CD8 | |
| --- | --- | --- | --- | --- | --- | --- |
|  | Mean | SE | Mean | SE | Mean | SE |
| CD44^+^ PD-1^+^ | 11.3 | 1.12 | 9.87 | 1.76 | 14.9 | 2.1 |
| CD44^+^ PD-1^-^ | 0.2 | 0.05 | 0.28 | 0.11 | 0.1 | 0.06 |

C. Spleen p21 (%)

|  | CD3 | | CD4 | | CD8 | |
| --- | --- | --- | --- | --- | --- | --- |
|  | Mean | SE | Mean | SE | Mean | SE |
| CD44^+^ PD-1^+^ | 0.74 | 0.08 | 0.62 | 0.09 | 2.54 | 0.33 |
| CD44^+^ PD-1^-^ | 0.07 | 0.02 | 0.1 | 0.04 | 0.12 | 0.04 |

D. H2AX MFI

|  | | CD3^+^ PD-1^-^ | | CD3^+^ PD-1^+^ | |
| --- | --- | --- | --- | --- | --- |
|  |  | Mean | SE | Mean | SE |
| Spleen | Young | 592 | 79 | 566 | 65 |
|  | Aged | 1158 | 90 | 969 | 45 |
| eVAT | Young | 2202 | 159 | 2682 | 109 |
|  | Aged | 1451 | 95 | 4235 | 221 |

Figure 3

B. PD-1^+^ MP T cells　(%) Spleen

|  | CD4^+^ T cells | | CD8^+^ T cells | |
| --- | --- | --- | --- | --- |
|  | Mean | SE | Mean | SE |
| 11W | 3.95 | 0.86 | 1.52 | 0.86 |
| 70W | 25.41 | 1.85 | 10.49 | 1.11 |
| 100W | 31.07 | 2.60 | 12.91 | 2.23 |

D. PD-1^+^ MP T cells (%) eVAT

|  | CD4^+^ T cells | | CD8^+^ T cells | |
| --- | --- | --- | --- | --- |
|  | Mean | SE | Mean | SE |
| 11W | 11.20 | 0.43 | 11.60 | 0.57 |
| 70W | 31.00 | 1.90 | 16.50 | 0.76 |
| 100W | 37.20 | 2.10 | 39.60 | 2.46 |

PD-1^+^ MP T cell number/g eVAT (×10^3^)

|  | CD4^+^ T cells | | CD8^+^ T cells | |
| --- | --- | --- | --- | --- |
|  | Mean | SE | Mean | SE |
| 11W | 11.27 | 2.88 | 7.08 | 1.95 |
| 70W | 42.44 | 10.52 | 28.58 | 8.45 |
| 100W | 76.93 | 9.40 | 53.91 | 10.49 |

Figure 4

A. Body weight (g)

| Time(weeks) | 51 | 55 | 59 | 63 | 67 | 71 | 75 | 79 | 83 | 87 |
| --- | --- | --- | --- | --- | --- | --- | --- | --- | --- | --- |
| AL Mean | 29.9 | 30.6 | 34.8 | 37.0 | 40.1 | 43.8 | 45.5 | 46.1 | 43.9 | 40.9 |
| AL SE | 0.80 | 1.09 | 1.63 | 1.91 | 2.42 | 2.89 | 3.10 | 3.31 | 2.99 | 2.80 |
| CR Mean | 32.4 | 32.3 | 26.7 | 28.0 | 29.4 | 28.5 | 26.6 | 28.0 | 27.8 | 27.5 |
| CR SE | 0.47 | 0.80 | 0.83 | 1.32 | 1.31 | 0.71 | 1.06 | 1.15 | 1.05 | 1.01 |

B. eVAT (mg)

|  | Mean | SE |
| --- | --- | --- |
| AL | 1484.5 | 107.0 |
| CR | 299.5 | 3.5 |

C. OGTT (mg/dl)

|  | 0 min | | 15 min | | 30 min | | 60 min | | 120 min | |
| --- | --- | --- | --- | --- | --- | --- | --- | --- | --- | --- |
|  | Mean | SE | Mean | SE | Mean | SE | Mean | SE | Mean | SE |
| AL | 94.0 | 9.79 | 326.8 | 22.72 | 283.8 | 20.44 | 187.3 | 18.51 | 137.5 | 7.24 |
| CR | 89.3 | 0.95 | 155.0 | 14.70 | 153.3 | 9.85 | 145.0 | 7.40 | 115.8 | 4.35 |

ITT (% change)

|  | 0 min | | 15 min | | 30 min | | 60 min | | 120 min | |
| --- | --- | --- | --- | --- | --- | --- | --- | --- | --- | --- |
|  | Mean | SE | Mean | SE | Mean | SE | Mean | SE | Mean | SE |
| AL | 100 | 0 | 66.2 | 7.0 | 49.5 | 3.9 | 48.9 | 5.9 | 66.7 | 6.2 |
| CR | 100 | 0 | 66.3 | 2.6 | 56.5 | 2.5 | 60.0 | 5.5 | 74.0 | 9.3 |

D. Insulin (ng/ml)

|  | Mean | SE |
| --- | --- | --- |
| AL | 0.50 | 0.03 |
| CR | 0.24 | 0.05 |

Adiponectin (μg/ml)

|  | Mean | SE |
| --- | --- | --- |
| AL | 15.74 | 2.49 |
| CR | 30.05 | 2.66 |

Leptin (ng/ml)

|  | Mean | SE |
| --- | --- | --- |
| AL | 6.61 | 1.12 |
| CR | 4.39 | 0.48 |

Figure 5

1. PD-1^+^ MP T cells (%) eVAT

|  | CD4^+^ T cells | | CD8^+^ T cells | |
| --- | --- | --- | --- | --- |
|  | Mean | SE | Mean | SE |
| AL | 39 | 2.1 | 34 | 0.45 |
| CR | 43 | 1.8 | 17 | 1.5 |

PD-1^+^ MP T cell number/g eVAT (×10^3^)

|  | CD4^+^ T cells | | CD8^+^ T cells | |
| --- | --- | --- | --- | --- |
|  | Mean | SE | Mean | SE |
| AL | 37.95 | 3.33 | 28.57 | 2.21 |
| CR | 5.12 | 0.73 | 4.42 | 0.64 |

D. PD-1^+^ MP T cells (%) Spleen

|  | CD4^+^ T cells | | CD8^+^ T cells | |
| --- | --- | --- | --- | --- |
|  | Mean | SE | Mean | SE |
| AL | 35 | 0.6 | 13 | 1.6 |
| CR | 22 | 0.45 | 2.1 | 0.34 |

F. Cell percentage (%)

|  | M/SVF | | M1/M | | M2/M | |
| --- | --- | --- | --- | --- | --- | --- |
|  | Mean | SE | Mean | SE | Mean | SE |
| AL | 14.5 | 1.8 | 22.3 | 1.7 | 53 | 3.1 |
| CR | 4.8 | 0.45 | 5.7 | 0.98 | 81 | 5.6 |

Cell number/eVAT (×10^3^)

|  | M | | M1 | | M2 | |
| --- | --- | --- | --- | --- | --- | --- |
|  | Mean | SE | Mean | SE | Mean | SE |
| AL | 177.4 | 11.0 | 41.6 | 3.2 | 100.1 | 4.3 |
| CR | 9.7 | 1.0 | 0.6 | 0.2 | 8.1 | 1.1 |

H. Adipocyte diameter (μm)

|  | Mean | SE |
| --- | --- | --- |
| AL | 106 | \| 4.7 \| \| --- \| |
| CR | 55 | \| 2.8 \| \| --- \| |

CLS number/LPF

|  | Mean | SE |
| --- | --- | --- |
| AL | 6.1 | 1.3 |
| CR | 1.2 | 0.7 |

1. Expression (AU)

|  | TNFα | | IL-6 | | CCL2 | | OPN | |
| --- | --- | --- | --- | --- | --- | --- | --- | --- |
|  | Mean | SE | Mean | SE | Mean | SE | Mean | SE |
| AL | 1.00 | 0.15 | 1.00 | 0.24 | 1.00 | 0.16 | 1.00 | 0.19 |
| CR | 0.44 | 0.09 | 0.30 | 0.11 | 0.44 | 0.02 | 0.22 | 0.10 |

Figure 6.

1. Food intake (g)

|  | IgG | | α-PD-1 | |
| --- | --- | --- | --- | --- |
|  | Mean | SE | Mean | Mean |
| Before | 3.77 | 0.10 | 3.75 | 0.16 |
| After | 3.77 | 0.08 | 3.76 | 0.13 |

1. Body weight (g)

|  | IgG | | α-PD-1 | |
| --- | --- | --- | --- | --- |
|  | Mean | SE | Mean | SE |
| Before | 35.38 | 0.78 | 35.45 | 1.04 |
| After | 35.96 | 0.72 | 35.86 | 0.88 |

1. eVAT (mg)

|  | IgG | | α-PD-1 | |
| --- | --- | --- | --- | --- |
|  | Mean | SE | Mean | SE |
| eVAT | 1004.0 | 69 | 974.7 | 109 |

1. OGTT (Before) (mg/dl)

|  | 0 min | | 15 min | | 30 min | | 60 min | | 120 min | |
| --- | --- | --- | --- | --- | --- | --- | --- | --- | --- | --- |
|  | Mean | SE | Mean | SE | Mean | SE | Mean | SE | Mean | SE |
| IgG | 94.0 | 2.9 | 309.8 | 15.2 | 202.8 | 6.8 | 146.3 | 10.5 | 110.0 | 7.6 |
| α-PD-1 | 92.5 | 2.3 | 300.8 | 28.8 | 212.8 | 9.0 | 137.8 | 5.4 | 112.0 | 3.6 |

OGTT (After) (mg/dl)

|  | 0 min | | 15 min | | 30 min | | 60 min | | 120 min | |
| --- | --- | --- | --- | --- | --- | --- | --- | --- | --- | --- |
|  | Mean | SE | Mean | SE | Mean | SE | Mean | SE | Mean | SE |
| IgG | 93.5 | 2.1 | 320.3 | 9.7 | 235.3 | 9.9 | 164.0 | 3.9 | 116.0 | 4.8 |
| α-PD-1 | 89.8 | 3.5 | 236.5 | 19.3 | 199.8 | 7.8 | 151.3 | 9.7 | 105.0 | 4.2 |

1. ITT (Before) (% change)

|  | 0 min | | 15 min | | 30 min | | 60 min | | 120 min | |
| --- | --- | --- | --- | --- | --- | --- | --- | --- | --- | --- |
|  | Mean | SE | Mean | SE | Mean | SE | Mean | SE | Mean | SE |
| IgG | 100 |  | 83.0 | 2.2 | 73.8 | 2.8 | 68.5 | 3.4 | 77.8 | 3.3 |
| α-PD-1 | 100 |  | 85.9 | 4.1 | 67.4 | 6.4 | 74.5 | 5.8 | 76.2 | 5.9 |

ITT (After) (% change)

|  | 0 min | | 15 min | | 30 min | | 60 min | | 120 min | |
| --- | --- | --- | --- | --- | --- | --- | --- | --- | --- | --- |
|  | Mean | SE | Mean | SE | Mean | SE | Mean | SE | Mean | SE |
| IgG | 100 |  | 86.9 | 5.7 | 79.6 | 6.5 | 78.5 | 6.5 | 78.0 | 6.9 |
| α-PD-1 | 100 |  | 78.5 | 4.7 | 58.1 | 7.1 | 56.4 | 10.3 | 64.7 | 7.3 |

1. Insulin (ng/ml)

|  | Mean | SE |
| --- | --- | --- |
| IgG | 0.46 | 0.04 |
| α-PD-1 | 0.33 | 0.05 |

Adiponectin (μg/ml)

|  | Mean | SE |
| --- | --- | --- |
| IgG | 11.14 | 1.76 |
| α-PD-1 | 18.45 | 2.70 |

Leptin (ng/ml)

|  | Mean | SE |
| --- | --- | --- |
| IgG | 7.38 | 1.71 |
| α-PD-1 | 4.54 | 0.77 |

Figure 7.

B. Percentage of Naïve T cells (%)

|  | Spleen | | eVAT | |
| --- | --- | --- | --- | --- |
|  | CD4 | CD8 | CD4 | CD8 |
| IgG Mean  SE | 7.73 | 11.62 | 6.94 | 3.47 |
|  | 0.54 | 1.08 | 0.70 | 0.51 |
| α-PD-1 Mean  SE | 20.42 | 24.83 | 13.13 | 13.60 |
|  | 1.75 | 2.158 | 1.10 | 0.76 |

Percentage of effect memory T cells (%)

|  | Spleen | | eVAT | |
| --- | --- | --- | --- | --- |
|  | CD4 | CD8 | CD4 | CD8 |
| IgG Mean  SE | 83.46 | 45.88 | 91.23 | 69.78 |
|  | 4.12 | 2.746 | 4.29 | 2.40 |
| α-PD-1 Mean  SE | 64.60 | 32.30 | 68.06 | 51.89 |
|  | 3.50 | 3.43 | 3.51 | 2.42 |

D. PD-1^+^ MP T cells (%)

|  | CD4^+^ T cells | | CD8^+^ T cells | |
| --- | --- | --- | --- | --- |
|  | Mean | SE | Mean | SE |
| IgG | 51.75 | 7.30 | 32.00 | 4.33 |
| α-PD-1 | 19.90 | 6.04 | 15.11 | 6.51 |

PD-1^+^ MP T cell number/g eVAT (×10^3^)

|  | CD4^+^ T cells | | CD8^+^ T cells | |
| --- | --- | --- | --- | --- |
|  | Mean | SE | Mean | SE |
| IgG | 36.93 | 7.39 | 20.45 | 7.68 |
| α-PD-1 | 2.17 | 0.38 | 1.90 | 1.00 |

F. PD-1^+^ MP T cells (%)

|  | CD4^+^ T cells | | CD8^+^ T cells | |
| --- | --- | --- | --- | --- |
|  | Mean | SE | Mean | SE |
| IgG | 28.05 | 0.95 | 9.05 | 2.02 |
| α-PD-1 | 14.64 | 1.88 | 4.31 | 0.62 |

1. Percentage (%)

|  | M1/M | | M2/M | |
| --- | --- | --- | --- | --- |
|  | Mean | SE | Mean | SE |
| IgG | 26.4 | 5.2 | 53.7 | 4.2 |
| α-PD-1 | 8.7 | 2.8 | 56.9 | 1.9 |

1. Adipocyte diameter (μm)

|  | Mean | SE |
| --- | --- | --- |
| IgG | 103.6 | 4.7 |
| α-PD-1 | 105.9 | 3.7 |

CLS number/LPF

|  | Mean | SE |
| --- | --- | --- |
| IgG | 5.56 | 0.86 |
| α-PD-1 | 3.17 | 0.36 |

1. Expression (AU)

|  | TNFα | | IL-6 | | CCL2 | | OPN | |
| --- | --- | --- | --- | --- | --- | --- | --- | --- |
|  | Mean | SE | Mean | SE | Mean | SE | Mean | SE |
| IgG | 1.00 | 0.15 | 1.00 | 0.24 | 1.00 | 0.16 | 1.00 | 0.19 |
| α-PD-1 | 0.76 | 0.11 | 0.45 | 0.02 | 0.32 | 0.08 | 0.66 | 0.17 |
